# The Role of MAPK3/1 and AKT in the Acquisition of High Meiotic and Developmental Competence of Porcine Oocytes Cultured In Vitro in FLI Medium

**DOI:** 10.3390/ijms222011148

**Published:** 2021-10-15

**Authors:** Radek Procházka, Alexandra Bartková, Lucie Němcová, Matej Murín, Ahmed Gad, Kateřina Marcollová, Veronika Kinterová, Andrea Lucas-Hahn, Jozef Laurinčík

**Affiliations:** 1 Laboratory of Developmental Biology, Institute of Animal Physiology and Genetics of the Czech Academy of Sciences, 27721 Liběchov, Czech Republic; alexandra.bartkova@ukf.sk (A.B.); Nemcova@iapg.cas.cz (L.N.); Murin@iapg.cas.cz (M.M.); Gad@iapg.cas.cz (A.G.); Marcollova@iapg.cas.cz (K.M.); Kinterova@iapg.cas.cz (V.K.); laurincik@gmail.com (J.L.); 2 Faculty of Natural Sciences, Constantine the Philosopher University in Nitra, 94901 Nitra, Slovakia; 3 Department of Animal Production, Faculty of Agriculture, Cairo University, Giza 12613, Egypt; 4 Friedrich-Loeffler-Institut, Institute of Farm Animal Genetics (ING), Höltystr. 10, Mariensee, 31535 Neustadt, Germany; andrea.lucas-hahn@fli.de

**Keywords:** FGF2, LIF, IGF1, oocyte maturation, oocyte competence, MAP kinase 3/1, AKT kinase, gene expression

## Abstract

The developmental potential of porcine oocytes cultured in vitro was remarkably enhanced in a medium containing FGF2, LIF and IGF1 (FLI) when compared to a medium supplemented with gonadotropins and EGF (control). We analyzed the molecular background of the enhanced oocyte quality by comparing the time course of MAPK3/1 and AKT activation, and the expression of genes controlled by these kinases in cumulus-oocyte complexes (COCs) cultured in FLI and the control medium. The pattern of MAPK3/1 activation in COCs was very similar in both media, except for a robust increase in MAPK3/1 phosphorylation during the first hour of culture in the FLI medium. The COCs cultured in the FLI medium exhibited significantly higher activity of AKT than in the control medium from the beginning up to 16 h of culture; afterwards a deregulation of AKT activity occurred in the FLI medium, which was not observed in the control medium. The expression of cumulus cell genes controlled by both kinases was also modulated in the FLI medium, and in particular the genes related to cumulus-expansion, signaling, apoptosis, antioxidants, cell-to-cell communication, proliferation, and translation were significantly overexpressed. Collectively, these data indicate that both MAPK3/1 and AKT are implicated in the enhanced quality of oocytes cultured in FLI medium.

## 1. Introduction

The substantial improvement in techniques for the maturation and fertilization of mammalian oocytes under in vitro conditions enabled the development of effective methods for assisted reproduction in human medicine and brought new approaches to animal breeding. Still, a great deal of work has to be carried out to clarify all aspects of oocyte biology so that its potential can be fully explored in IVF programs and other reproductive biotechnologies. Among the crucial steps of oocyte biology that need further research are the mechanisms regulating the final maturation of the oocytes, i.e., the release of the fully grown oocyte from the natural block in meiotic prophase, and its progression up to metaphase II (MII). In vivo, the maturation of oocytes is stimulated by the preovulatory surge of the luteinizing hormone (LH). The LH binds to LH receptors on mural granulosa cells and generates the production of specific epidermal growth factor-like peptides that transmit signals to the cumulus cells and the oocyte itself [1]. The interaction and feedback loops among these follicular cells result in the resumption of meiosis in oocytes, expansion of the cumulus cells (CCs), ovulation of the matured oocyte-cumulus complex (COC) into the oviduct, and luteinization of the granulosa cells [1,2].

Work to mimic the preovulatory events in in vitro culture systems resulted in designing novel, more efficient, but also more complicated techniques for the culture of mammalian oocytes, such as delaying the spontaneous maturation of oocytes by chemical or natural inhibitors (two-step cultures) [2], simulated physiological oocyte maturation [3] or the sequential exposure of oocytes to different concentrations of steroid hormones and gonadotropins [4]. One of the efficient but still undemanding approaches is the supplementation of culture media with growth hormones/cytokines that are naturally produced by cumulus/granulosa cells during final follicle development. In mammals, the gonadotropins are commonly combined with epidermal growth factor (EGF) or EGF-like factors that are produced by follicular cells. This combination proved to enhance the maturation as well as the developmental competence of pig, cattle, sheep, horse, cat, dog, rodent, monkey as well as human oocytes [5]. In the same vein, a significant improvement in maturation and developmental competence of mammalian oocytes occurred after the supplementation of the culture medium with several other growth factors/cytokines. One of the growth factors which seems to play an important role in vitro maturation is fibroblast growth factor 10 (FGF10) which was discovered at the mRNA and protein levels in bovine oocyte, theca and granulosa cells [6]. There is increasing evidence that the supplementation of culture media with FGF10 not only supports the oocyte maturation rate, but also enhances cleavage, development into blastocysts and the number of cells at the blastocyst stage in pigs and ruminants [7,8,9,10]. An enhanced nuclear maturation, an increase in cumulus cell survival and extracellular matrix quality were observed in bovine COCs cultured in a medium supplemented with another member of the FGF-family of growth factors-FGF2 [11]. It has been convincingly demonstrated that insulin-like growth factor 1 (IGF1) is the component of serum that enables the expansion of porcine CCs in response to FSH [12] by a phosphoinositide-3-kinase/v-akt murine thymoma viral oncogene homolog (PI3K/AKT) and mitogen-activated kinase 3/1 (MAPK3/1)-dependent mechanism [13]. Further studies indicated that IGF1 can also improve oocyte maturation rate and quality and consequently enhance embryo development and blastocyst formation [14,15,16,17]. Porcine and bovine oocyte nuclear maturation and blastocyst development were also enhanced in medium supplemented with leukemia inhibitory factor (LIF); moreover it was shown that LIF phosphorylates MAPK3/1 and STAT3 in the oocytes, highlighting the importance of LIF/STAT3 pathways during in vitro maturation [18,19]. Bovine COCs treated with LIF during in vitro maturation exhibited an increased expression of miR-21 in both oocytes and CCs when compared to immature oocytes [20]. Furthermore, LIF improved the blastocyst hatching rate in bovines and proved to be a potential regulator of miRNAs expression in oocytes and CCs, as well as gene expression in early embryos [20]. A combination of growth factors and cytokines, namely FGFs, IGF1, IGF2 and LIF, enhanced the developmental potential of bovine and buffalo embryos and increased the quality of hatched bovine blastocysts in vitro [21,22].

A remarkable improvement in maturation and developmental competence has been recently achieved in porcine oocytes cultured under in vitro conditions. The supplementation of traditional medium containing gonadotropins (FSH, LH) and EGF by three additional cytokines, FGF2, LIF and IGF1 (FLI medium) enabled the removal of undefined sources of proteins, such as fetal calf serum or follicular fluid, and their replacement with polyvinyl alcohol or bovine serum albumin (BSA), without loss of maturation competence. On the contrary, such a modification of the traditional culture medium resulted in a conspicuous increase in the quality of porcine oocytes, which was manifested by (1) an equal or higher developmental competence of gilt-derived oocytes cultured in FLI medium compared to sow-derived oocytes cultured in the traditional medium [23], (2) a higher quality of blastocysts produced from gilt oocytes cultured in DMEM and FLI medium [24] and (3) quadrupling efficiency in the production of genetically modified pigs derived from oocytes cultured in FLI compared to the traditional (control) medium [25].

The essential hormonal and growth factor/cytokine components of the FLI medium are known to activate a variety of signaling pathways in follicular somatic cells, of which the PI3K/AKT and MAPK3/1 pathways appear to cause the most significant impact on events associated with the regulation of oocyte meiosis and ovulation.

The activation of MAPK3/1 in preovulatory follicles proved to be essential for the termination of granulosa cell proliferation and for the induction of genes controlling cumulus expansion, luteinization and ovulation [26]. It has been demonstrated in mice and pigs that MAPK3/1 activity in CCs is essential for the gonadotropin-induced resumption of oocyte meiosis, the stimulation of cumulus expansion and enhanced expression of expansion-related genes (*HAS2* and *PTGS2*) [27,28,29,30,31]. In contrast, the activation of MAPK3/1 in the oocyte does not seem necessary for the resumption of meiosis since it occurs at the time of or even after germinal vesicle breakdown (GVBD) [32] and specific inhibition of MAPK3/1 activity does not prevent GVBD in mouse and bovine oocytes [33,34]. Nevertheless, MAPK3/1 signaling participates in formation of the meiotic spindle, metaphase I/metaphase II transition and metaphase II arrest in mammalian oocytes [34,35]. Yuan et al. [25] expressed an assumption that the different kinetics of MAPK3/1 activation in the CCs may be the basis of the enhanced quality of the oocytes cultured in FLI medium. In that study, the proportions of activated and total MAPK3/1 remained unchanged in COCs cultured in the control medium over the period of 22 h and increased at the end of maturation at 42 h. In contrast, the activity of MAPK3/1 in COCs cultured in FLI medium dropped at 2 h of culture and increased thereafter with a maximum activity at 22 h and sharply decreased at the end of culture. We have shown previously that MAPK3/1 can be rapidly activated in pig COCs by EGF or by a ligand-independent mechanism involving SRC or/and protein kinase C activities [36]. The significance of this rapid activation of MAPK3/1, occurring by 1 h after the onset of maturation, for the promotion of oocyte quality has not been studied so far. This prompted us to analyze in detail the activation of MAPK3/1 in porcine COCs cultured in FLI medium and to compare it with the course of MAPK3/1 activation in the control medium.

There is a large body of evidence in the literature to suggest that, besides MAPK3/1, the AKT also affects metabolism, proliferation and survival of somatic follicular cells and plays a significant role in the regulation of special follicular functions such as the resumption of oocyte maturation, expansion of CCs and ovulation [32,37,38]. In CCs, AKT is preferentially involved in the regulation of cell proliferation and survival with a modest effect on cumulus expansion [37]. In porcine CCs, PI3K/AKT signaling is involved in the regulation of intercellular communication by connexin 43 phosphorylation [39] and in the promotion of FSH-stimulated synthesis and retention of hyaluronic acid in porcine COCs [13,40]. Further studies revealed that AKT activity is required for a high expression of *HAS2* and *TNFAIP6*, the key enzymes involved in the production and stabilization of hyaluronic acid (HA) in the expanding cumulus [31,41,42]. However, AKT does not seem to be involved in meiosis resumption in porcine oocytes, since AKT inhibitors do not prevent GVBD under in vitro conditions. Nevertheless, AKT is essential for the completion of meiosis since the inhibitors stop meiosis at the MI stage [31,43]. It is not known whether the growth factors/cytokines of the FLI medium change the extent and the dynamics of AKT activation in COCs, and the significance of these potential changes for promotion of oocyte maturation and developmental competence has not been studied so far.

We suppose that the promotion of meiotic and developmental competence of porcine oocytes cultured in FLI medium compared to the traditional medium may be caused by specific patterns of MAPK3/1 and AKT activation in the COCs, and by the differential expression of genes affecting periovulatory events. In this work we compared the maturation and developmental competence of oocytes cultured in the control medium, supplemented with gonadotropins and EGF, and in the complete FLI medium. In addition, we assessed the extent and temporal pattern of MAPK3/1 and AKT activation during the culture of COCs in both media. Finally, we studied the expression patterns of key genes of the CCs which are regulated by MAPK3/1 and AKT signaling and which are required for cumulus expansion, signaling, apoptosis regulation, proliferation, cell communication and/or have proved to be markers of oocyte developmental competence.

## 2. Results

### 2.1. FLI Cytokines Promote the Maturation and Developmental Capacity of Porcine Oocytes

The COCs cultured in basal M199 medium supplemented with BSA did not undergo expansion and the oocytes remained mostly in the germinal vesicle (GV) stage; about 9% of oocytes stopped at the GVBD stage and only 27% of the oocytes completed maturation to the MII stage (Table 1). The addition of gonadotropins (PMSG+hCG) to the basal medium stimulated the expansion of CCs, but surprisingly it did not change the proportions of maturing oocytes, which indicates that in serum-free medium the addition of growth factors is essential for the successful maturation of porcine oocytes. This indication was confirmed by the addition of EGF to the gonadotropin-supplemented medium (PMSG+hCG+EGF), which significantly increased the proportion of matured oocytes to 70%. This type of medium served in further experiments as the control medium. Further significant improvement of oocyte maturation was achieved by the addition of 3 growth factors/cytokines (FGF2, LIF, IGF1) to the control medium, since almost 98% of oocytes reached the MII stage at the end of the culture.

In the next experiment, we assessed the effect of individual components of FLI medium on the maturation of oocytes. As shown in Table 2, each of the 3 additional growth factors significantly improved the maturation of oocytes to MII stage compared to the control medium. The differences between the individual growth factors were not significant, nevertheless, there was a clear tendency in all 3 replicates of this experiment that LIF and complete FLI are even more efficient than FGF2 or IGF1.

Next, we considered whether the course of oocyte maturation is different in the control and FLI medium, since this knowledge might be important for further analyzes. Specifically, we concentrated our attention on the time at which GVBD occurs. For this reason, the oocytes were cultured for 16 and 24 h; a group of oocytes was cultured to the MII stage in each replicate as a biological control. The data presented in Table 3 document that the maturation of oocytes in both types of medium was synchronous until 16 h of culture, but beyond this point the progression of meiosis significantly accelerated in FLI medium.

Finally, we compared the developmental competence of oocytes cultured in the control and FLI medium by assessing their parthenogenetic development after ionophore activation (Table 4). The oocytes cultured in FLI medium exhibited a significantly higher cleavage rate on Day 2 of culture and blastocysts rate on Day 6 than their counterparts cultured in the control medium.

### 2.2. Time Course of MAPK3/1 Activation in COCs Cultured in Control and FLI Medium

The activation of MAPK3/1 was first assessed over a short-term culture period (0–4 h) and then, over a long-term culture period (0–40 h). The reason for this approach was that we recently identified two different mechanisms of MAPK3/1 activation in porcine CCs by gonadotropins: the first one relies on a rapid ligand-independent activation of EGF-receptor (EGFR) requiring SRC and protein kinase C activities, and the second mechanism is the ligand-dependent activation of EGFR, requiring the synthesis of EGF-like peptides [36]. Besides the gonadotropins, both control and FLI media contain EGF that rapidly activates MAPK3/1 via direct binding to EGFR [36]. The speed with which LIF, IGF1 and FGF2 activate or potentiate activation of MAPK3/1 in CCs has not yet been precisely assessed.

Despite the fact that we were interested in the kinase activity in CCs, we analyzed samples prepared from whole COCs for two reasons: (1) the signal provided by the oocyte itself is negligible when compared to the signal provided by the cumulus compartment, as shown below, and (2) stress associated with CCs removal may affect the activity of the kinase.

**Figure 1:**
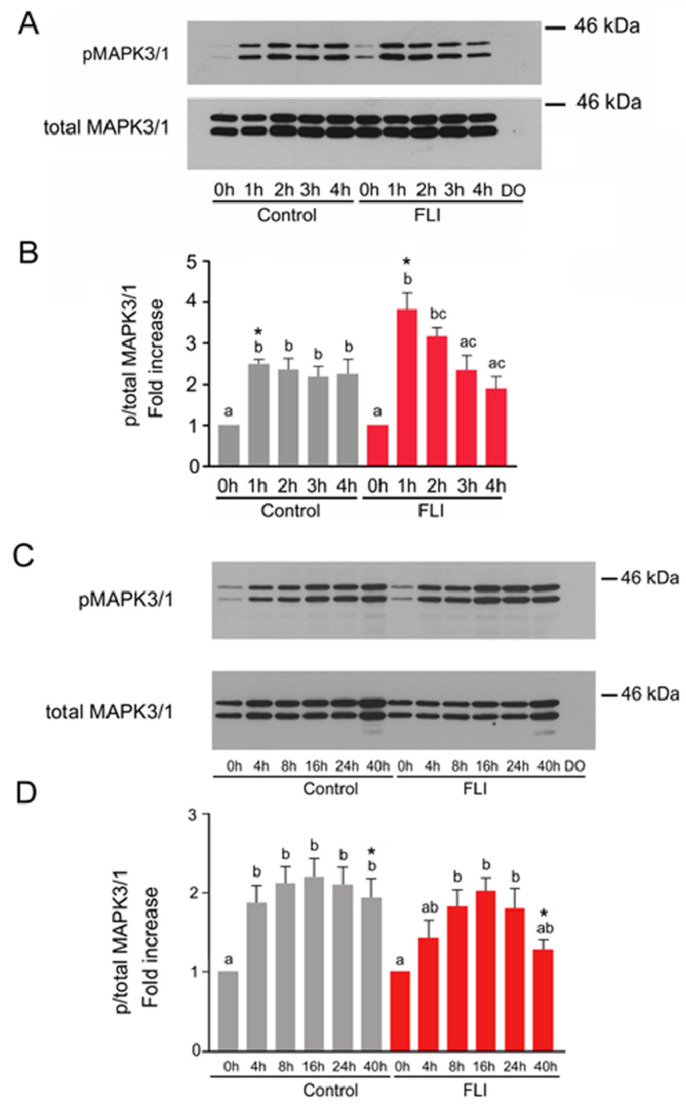
Time course of MAPK3/1 activation in COCs cultured in control and FLI medium. Activation of MAPK3/1 in control and FLI medium during short-term (**A**) or long-term (**C**) culture. The panels show representative results of immunoblotting of phosphorylated and total MAPK3/1 in samples of 25 COCs cultured in vitro for the indicated periods of time. Denuded oocytes (DO) originating from COCs cultured for 4 h or 40 h were used for assessing their contribution to the entire COCs signal. Quantification of the activated MAPK3/1 in three independent experiments was performed by densitometry and is shown in the graphs as proportions of phosphorylated and total MAPK3/1, and expressed in arbitrary units as the fold increase over the proportion found in unstimulated COCs at the beginning of culture (**B**,**D**). The different superscripts or superscripts with no common letter above the columns indicate significant differences within the same treatment. Asterisks above the columns indicate a significant difference between the treatments at the same time of culture (*p* < 0.05).

The activation of MAPK3/1 in COCs cultured in the control medium, documented by a significant increase in the relative amount of the phosphorylated kinases compared to the level in COCs before culture, occurred during the first hour of culture and remained high for 4 h. The activation of MAPK3/1 in COCs cultured in the FLI medium also occurred during the first hour of culture and was significantly higher than in the COCs cultured in the control medium at this time (Figure 1A,B). Afterwards, the activity of MAPK3/1 in COCs cultured for 2–4 h in FLI medium gradually decreased on levels comparable with those found in COCs cultured in the control medium. The signal of phosphorylated and total MAPK3/1 provided by 25 denuded oocytes originated from COCs cultured for 4 h was not detectable under the same conditions, which indicates that the obtained data exclusively reflect the activity of MAPK3/1 in CCs.

During the long-term culture, the increased activity of MAPK3/1 in COCs cultured in the control medium was detected at 4 h, reached its maximum at 16 h and remained stable by the end of culture at 40 h. In concert with the data obtained during the short-term culture, the significantly increased activity of MAPK3/1 was first detected in the COCs cultured in FLI medium at 8 h of culture, reached maximum at 16 h of culture, remained high at 24 h and dropped at 40 h of culture, when it was significantly lower than in COCs cultured in control medium. There was no difference in the relative increase in MAPK3/1 phosphorylation between the control and FLI medium until 24 h of culture (Figure 1C,D).

Collectively, the pattern of MAPK3/1 activation in COCs cultured in control and in FLI medium was very similar. The significant differences between both types of media were a robust increase in MAPK3/1 phosphorylation during the first hour of culture in FLI medium and a deregulation of MAPK3/1 activity in FLI medium at the end of culture.

### 2.3. Time Course of AKT Activation in COCs Cultured in Control and FLI Medium

**Figure 2:**
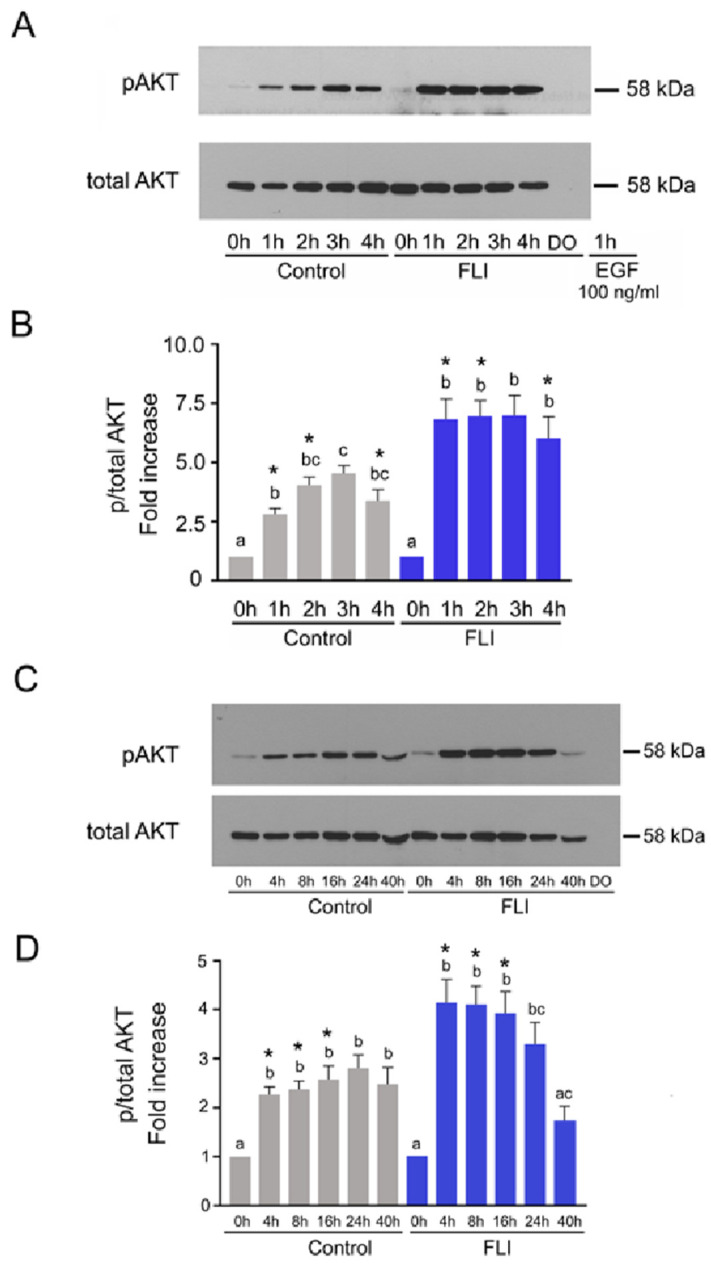
Time course of AKT activation in COCs culture in control and FLI medium. Activation of AKT in control and FLI medium during short-term (**A**) or long-term (**C**) culture. The panels show representative results of immunoblotting of phosphorylated and total AKT in samples of 25 COCs cultured in vitro for the indicated periods of time. Denuded oocytes (DO) cultured for 4 or 40 h were used for assessing their contribution to the entire COC signal. Quantification of the activated AKT in three independent experiments was performed by densitometry and is shown in the graphs as proportions of phosphorylated and total AKT, and expressed in arbitrary units as the fold increase over the proportion found in unstimulated COCs at the beginning of the culture (**B**,**D**). The different superscripts or superscripts with no common letter above the columns indicate significant differences within the same treatment. The asterisks above the columns indicate a significant difference between the treatments at the same time of culture (*p* < 0.05).

The activation of AKT occurred in the COCs cultured in the control medium during the first hour, further increased at 2 h, reached its maximum at 3 h and remained high by the end of the short-term culture. The phosphorylation of AKT in COCs cultured in FLI medium also occurred during the first hour and remained high for the entire culture period, but it was about 50 % higher than that found in the control medium at all the selected culture times (Figure 2A,B).

During the long-term culture of COCs in the control medium, a significantly increased phosphorylation of AKT was detected from 4 to 40 h, whereas in COCs cultured in FLI, the significant increase in AKT phosphorylation was observed between 4–24 h of culture with a rapid decrease at 40 h. The relative phosphorylation of AKT in COCs cultured in FLI was significantly higher than in COCs cultured in the control medium between 4–16 h. Again, the signal of phosphorylated and total AKT provided by 25 denuded oocytes originated from COCs cultured for 40 h was not detectable under the same experimental conditions, which indicates that the obtained data exclusively reflect the activity of the kinase in CCs (Figure 2C,D).

Collectively, the COCs cultured in FLI medium exhibited a significantly higher activity of AKT than COCs cultured in the control medium up to 16 h of culture. The patterns of AKT activation further differed between the two treatments in that a rapid deregulation of AKT activity occurred beyond 24 h of culture in FLI medium, which was not detected in COCs cultured in the control medium.

### 2.4. Expression of Cumulus Expansion and Signaling Related Genes in COCs Cultured in Control and FLI Medium

**Figure 3:**
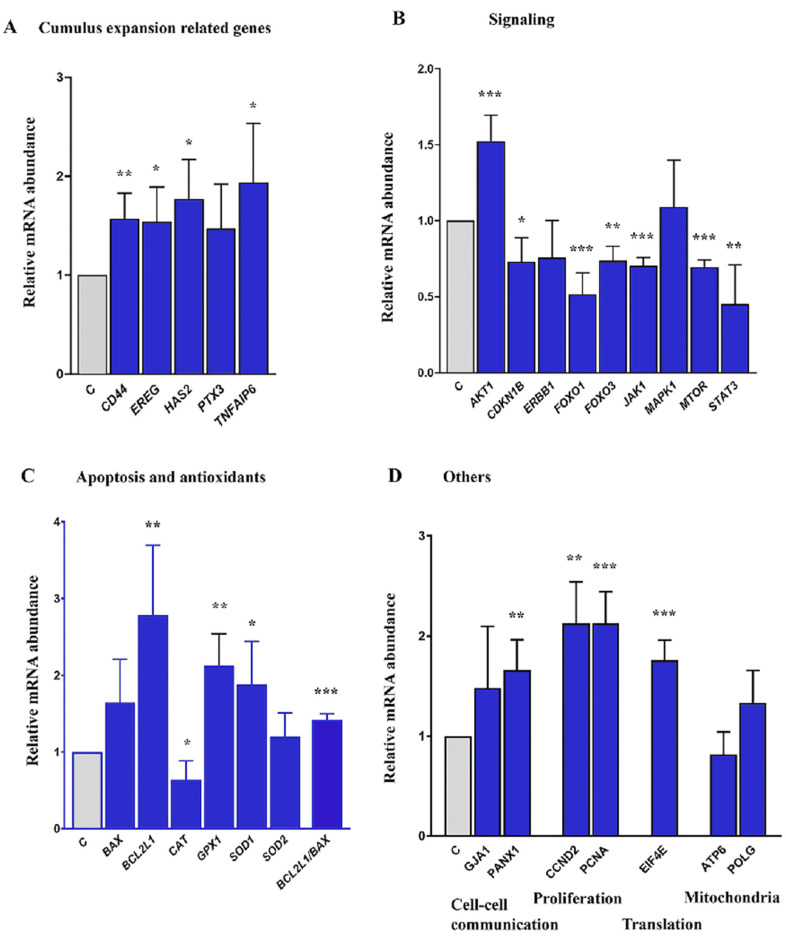
Gene expression in cumulus cells (CCs) cultured in control and FLI medium. Cumulus expansion-related genes (**A**), signaling pathways-related genes (**B**), genes related to apoptosis and antioxidants (**C**), and others (**D**). Differences are expressed in arbitrary units as the fold increase over the proportion found in CCs cultured in control (**C**). Asterisks above the columns indicate significant differences between the treatments at the same time of culture (*p* < 0.05 for single asterisk; *p* < 0.01 for double asterisk, *p* < 0.001 for triple asterisk).

In CCs, the addition of 3 growth factors/cytokines (FGF2, LIF, IGF1) significantly increased the expression of cumulus expansion-related genes (*CD*44, *EREG, HAS*2, and *TNFAIP*6) mRNA in comparison to the control medium. However, no differences were found for *PTX3* transcript. (Figure 3A). The transcript level of the signaling pathway gene *AKT1* was significantly higher in CCs cultured in FLI medium (Figure 3B). On the other hand, *CDKN*1*B, FOXO*1*, FOXO*3*, JAK1, MTOR*, and *STAT*3 were significantly downregulated and both *ERBB*1 and *MAPK*1 mRNAs were detected at the same level (Figure 3B). FLI medium significantly increased the expression of the antiapoptotic gene *BCL*2*L*1 and also transcripts for antioxidant proteins GPX1 and SOD1. The transcript level for *CAT* was significantly lower and that for *SOD*2 was expressed at the same level (Figure 3C). Transcripts for cell-to-cell communication (*PANX*1) and proliferation *(CCND*2 and *PCNA*) and translation-related (*EIF*4*E*) genes were also significantly overexpressed in FLI medium. No differences were detected for the mitochondria-related genes *ATP*6 and *POLG* (Figure 3D).

## 3. Discussion

The presented data confirmed that FLI medium promotes standards in the culture of porcine oocytes in vitro on a level comparable with in vivo conditions and clearly demonstrated that this is due to the action of three additional growth hormone/cytokines—FGF2, LIF and IGF1. A remarkably low maturation rate was achieved in oocytes cultured in the basal M199 medium supplemented with BSA and gonadotropins—only 26% of oocytes completed maturation to the MII stage, which was no improvement compared to the medium without gonadotropins. This indicates that in this type of culture medium, lacking serum or follicular fluid supplement, the addition of exogenous growth factors is essential for the proper stimulation of signaling pathways that regulate the resumption and completion of oocyte meiosis. Indeed, the addition of EGF to the M199 medium with BSA and PMSG/hCG increased the maturation rate to 70% and further improvement was achieved in the FLI medium, where more than 97% of cultured oocytes matured to MII stage. In a paper published previously, an additive effect of FLI cytokines on the maturation of porcine oocytes was reported [25]. In our experiments, the addition of each of the FLI cytokines to the control medium increased the maturation rate to over 90%, which was not significantly different from maturation in the complete FLI (96%). Nevertheless, in all replicates of this experiment, the maturation of oocytes in FLI medium was slightly better than in other groups, which makes the use of all three FLI growth factors/cytokines justified and advisable. The assessment of the parthenogenetic development of oocytes cultured in the control and FLI medium clearly showed that the latter medium not only stimulates better nuclear maturation, but it also enhances cytoplasmic maturation and oocyte developmental competence. Taken together, the introductory experiments of our study confirmed that this is an efficient and relatively simple model of significantly promoted oocyte meiotic and developmental competence, suitable for analyzing the molecular background of the observed biological features.

In our study, we found a prompt increase in MAPK3/1 activity in COCs cultured in both media during the first hour of culture: the amount of phosphorylated MAPK3/1 more than doubled in COCs cultured in the control medium and more than tripled in COCs cultured in FLI medium. The higher and faster phosphorylation of MAPK3/1 in FLI medium was undoubtedly caused by stronger stimulation of COCs due to the presence of the additional growth factors LIF, IGF1 and FGF2 which all possess the potential to activate MAPK3/1 in various cell types including CCs [44,45,46,47,48,49], or to potentiate the activation of MAPK3/1 induced by gonadotropins [13]. The next pattern of MAPK3/1 phosphorylation in FLI medium was similar to that described previously [25]— it dropped to the base level at 3 and 4 h and increased thereafter with a maximum at 24 h of culture and decreased at the end of culture at 40 h. The pattern of MAPK3/1 phosphorylation in COCs cultured in control medium was very similar to that observed in FLI medium with one exception—the significant increase in phosphorylation was noted throughout the culture period, including the last time point of assessment at 40 h. This feature is probably related to a slower pace of oocyte maturation and cumulus expansion in COCs cultured in the control medium. In summary, the only difference in the pattern of MAPK3/1 phosphorylation in COCs cultured in both types of media, which may affect the quality of oocytes, is its prompt and robust increase in the COCs cultured in FLI medium, which occurred soon after the onset of maturation and was significantly higher than in the control medium.

Such a prompt increase in MAPK3/1 activity may have occurred via the EGFR activation by a ligand-dependent or ligand-independent pathway [50]. The classical ligand for EGFR (ERBB1) present in FLI medium is EGF. However, the comparison with the control medium indicates that there are other mechanisms activated in CCs by the additional components of the FLI medium that contribute to the rapid activation of MAPK3/1. We have recently demonstrated that the rapid activation of the EGFR/MAPK3/1 pathway occurs in porcine CCs via a ligand-independent mechanism involving SRC and PKC activities [36]. In addition, each of the three FLI growth factors/cytokines possesses the potential for rapid MAPK3/1 activation. LIF binds to its specific membrane receptor and, in association with a glycoprotein GP130, mediates phosphorylation of several Janus kinases (JAKs) [44]. The phosphorylated JAKs then recruit the adaptor protein GRB2, which consequently recruits the guanine nucleotide exchange factor SOS. The recruited SOS activates the RAS GTPase, which then activates the canonical MAPK3/1 pathway [45,46]. Similarly, FGF2 binding to FGF-receptor heparan sulfate proteoglycan (HS) as a cofactor induces the formation of the ternary FGF2-FGFR-HS complex, which activates the FGFR intracellular tyrosine kinase domain by phosphorylating specific tyrosine residues [47,48]. The major FGFR kinase substrate, FRS2α, recruits the adaptor protein GRB2, which through SOS leads to activation of the MAPK pathway. Finally, the binding of IGF1 to IGFR leads to the phosphorylation of intracellular domain insulin receptor substrate-1 and SHC, which recruits the adaptor and exchange factors (GRB2/SOS) responsible for activating the MAPK cascade [49].

The physiological significance of this rapid MAPK3/1 activation in the porcine COCs is not clear. In general, in terms of the regulation of mammalian oocyte maturation, MAPK3/1 is involved in the regulation of the transport of meiosis-arresting cyclic nucleotides from granulosa/CCs to the oocyte through modulating gap junction permeability, regulating gene expression in CCs, including genes involved in the steroidogenesis and synthesis of extracellular matrix protein [32]. We assume that the rapid activation of MAPK3/1 is most important for the rapid induction of AREG peptide [51], which then serves as a ligand for EGFR and triggers a series of phosphorylation events resulting, among other things, in activation of the MAPK3/1 pathway [51,52]. The regulatory loop between MAPK3/1 and AREG is of crucial importance for the maintenance of EGFR in a phosphorylated state, which is a unique feature of somatic cells in the preovulatory follicle, essential for oocyte maturation and cumulus expansion [53,54]. In addition, the pattern of EGFR phosphorylation evoked by AREG and epiregulin (EREG) strongly affects the acquisition of high developmental competence of mammalian oocytes during the final stages of maturation [5,55]. It should be noted here that, in our experiments, FLI medium promoted fast *EREG* mRNA up-regulation in CCs in comparison to the control. On the other hand, we did not detect differences in the expression of *EGFR (ERBB*1) between control and FLI medium, which is in agreement with our previous results showing that the high activity of the EGFR in porcine cumulus cells is not determined by an increase in mRNA expression, but instead by post-transcriptional events [36,56].

The differences between control and FLI medium in the pattern of COCs AKT activation were more conspicuous than those observed in MAPK3/1. The relative increase in AKT phosphorylation was much higher in COCs cultured in FLI for most of the culture period, except at the end of culture, when a rapid deregulation of AKT activity occurred, which was not detected in COCs cultured in the control medium. There is a large body of evidence concerning the association of AKT activity in CCs with the developmental competence of mammalian oocytes, and the involvement of specific genes controlled by PI3K/AKT kinase signaling in the regulation of various events in preovulatory follicles and in cultured COCs. The addition of the PDK1/PI3K/AKT activator PS48 into the culture medium increased the expansion of cumulus cells and the maturation rate of porcine oocytes [57]. Vice versa, PI3K/AKT inhibitors LY294002 and SH6 reduced the cumulus expansion and developmental competence of porcine and bovine oocytes assessed by blastocyst rate after parthenogenetic activation [38,58,59]. These phenotype changes were accompanied by substantial changes in the expression of specific genes in CCs as well as in mRNA abundance in the oocytes. The stimulation of AKT signaling led in matured oocytes to an increased mRNA abundance of genes involved in cell signaling and proliferation, such as *cyclin B*1, *MOS*, *BMP*15, *GDF*9 and *CDC*2, and to a decreased expression of pro-apoptotic genes such as *BAX*, *BCL*2 and *caspase-*3 [57]. Similarly, a melatonin-reversed inhibition of AKT by SH6 was associated in bovine COCs with an up-regulation of genes involved in cell signaling, mitochondrial function and cumulus expansion (*GDF*9, *BMP*15, *ATPase*, *ATP5F*1*E*, *HAS*2, *TNFAIP*6 and *PTGS*2) and downregulation of the pro-apoptotic genes [59]. However, in human CCs obtained by dissection from aspirated matured COCs, a down-regulation of 11 genes controlled by PI3K/AKT and involved in proliferation and survival was closely associated with the high developmental competence of the donor oocytes assessed by a positive IVF outcome [60]. The authors further supported this conclusion by assessing several of these genes, namely *AKT*1, *Bcl*2*l*1 and *Shc*1, on the model of aging mouse oocytes. The results of this experiment revealed that the deregulation of these genes occurred at the optimal age of oocytes for fertilization, i.e., between 12–15 h post hCG application and was followed by increased expression in young and aged oocytes, respectively [60]. Therefore, it appears that the expression pattern of genes controlled by the PI3K/AKT pathway in cumulus/granulosa cells is time-specific. In cultured COCs, the initial overexpression of such genes in the early stages of maturation is followed by their deregulation, which is associated with terminal differentiation of the CCs associated with their expansion and luteinization. The decline in AKT activity at the end of the culture period, observed in our experiments in COCs cultured in FLI medium but not in control medium, is in concert with the described concept. At the mRNA level, we found that the expression of *AKT*1 was significantly higher in CCs cultured for 8 h in the FLI medium than in their counterparts cultured in the control medium. Signaling via the PI3K/AKT pathway can control several cellular processes, including proliferation, differentiation, and survival. Indeed, we detected the up-regulation of genes related to proliferation (*CCND*2 and *PCNA*), translation (*EIF*4*E*), and anti-apoptotic processes (*GPX*1*,* and *SOD*1*).* The FLI medium further promoted the up-regulation of anti-apoptotic gene *BCL2L1* mRNA expression. Moreover, genes related to cell-to-cell interactions (*PANX*1) were also up-regulated. Further, the higher expression of cumulus expansion-related genes *HAS*2, and *TNFAIP6* in CCs cultured for 4 h in the FLI medium correlated with the higher AKT activity, which is needed for transcription of these cumulus-expansion related genes [38,41]. HA-binding receptor CD44 is expressed at the membrane of CCs where the HA anchors. We confirmed that the expression of *CD44* and *HAS2* mRNAs was positively correlated.

An important role in the transcription of the genes under the control of the PI3K/AKT pathway are Forkhead box O (FOXO) transcription factors, namely FOXO1 and 3, which are known to inhibit cell growth and/or apoptosis signaling [61]. FOXOs are downstream targets of AKT kinase; upon activation, AKT phosphorylates FOXOs and creates a docking site for 14-3-3 protein. The binding of 14-3-3 to FOXO excludes FOXO from the nucleus and decreases the transcription of the genes controlled by FOXO [62]. Such inhibition of the transcriptional functions of FOXOs contributes to cell survival, growth and proliferation. Our data suggest that the inhibition of FOXOs may also occur at the transcription level, since after 8 h of culture of COCs, both *FOXO*1 and *FOXO*3 mRNAs significantly dropped in CCs in the FLI medium in comparison to the control medium. In addition, the FLI medium promotes the up-regulation of anti-apoptotic gene *BCL2L1* mRNA expression. Even though the transcript level for *BAX* also increased, the ratio of *BCL2L*1*/BAX* increased in CCs cultured in FLI medium. Moreover, we detected a significantly lower level of *CDKN*1*B* mRNAs in those CCs cultured in FLI medium. CDKN1B is an inhibitor of cyclin-dependent kinases and its activity is negatively associated with cell cycle progression. All these data support the opinion that the FLI medium stimulates proliferation, survival and an antiapoptotic environment in cultured COCs, at least during the first half of the culture period. The higher transcript abundance of *CDKN*1*B, FOXO*1 and *FOXO*3 mRNAs in CCs cultured in the control media with EGF alone may be associated with lesser cumulus expansion of COCs.

## 4. Materials and Methods

All chemicals were purchased from Sigma-Aldrich (Munich, Germany) unless otherwise stated. All plastic materials were purchased from Nunc (Roskilde, Denmark).

### 4.1. Culture Media and Reagents

The COCs were cultured either in control or modified FLI medium [24,25]. The composition of the control and FLI medium is given in Table 5.

### 4.2. Collection and Culture of Cumulus-Oocyte Complexes

Ovaries were obtained from premature gilts slaughtered at a local abattoir, their ovaries excised and transported to the laboratory in a thermo-flask at 38 °C. The contents of medium-size antral follicles about 3–6 mm in diameter were aspirated with a syringe connected to a 20 G needle, pooled in a test-tube and allowed to sediment for 10 min. The sediment was washed twice with PXM-Hepes [63], placed in a Petri dish, and the COCs were collected with a pipette. Only COCs surrounded by a compact multi-layered cumulus were selected for experiments. Groups of 25 or 50 COCs were cultured for 40–44 h in four-well dishes in 0.5 mL of culture medium at 38.5 °C in a humidified atmosphere of 5% CO_2_.

### 4.3. Assessment of Oocyte Maturation

To assess their nuclear maturation, oocytes were stripped of CCs by vortexing. The degenerated oocytes (<5%) were excluded from the evaluation and the remaining ones were mounted on slides and fixed in an acetic acid-ethanol solution (1:3) for 48 h. Oocytes were then stained with 1% orcein and observed with a light microscope. Oocytes were scored for GV, GVBD (mostly comprising oocytes at the metaphase I stage and few oocytes at late diakinesis, anaphase I or telophase I) and for metaphase II stage (MII).

### 4.4. Parthenogenetic Activation and Culture of Embryos

Parthenogenetic development rather than in vitro fertilization (IVF) is now often used for assessing the cytoplasmic maturation of cultured porcine oocytes since mammalian parthenogenetic embryos undergo normal preimplantation development [64]. In addition, this approach avoids the problems with polyspermy and semen variability associated with pig IVF in vitro. For that, CCs were removed from COCs by pipetting and washed twice in PXM-HEPES. Oocytes were activated by exposure to 10 µM ionomycin in PXM-HEPES for 5 min, washed twice in porcine zygote medium 3 (PZM 3) [65] supplemented with 2 mM 6-DMAP, and cultured for 5 h at 38.5 °C under a 5% CO_2_ atmosphere. Around 50 putative parthenotes were washed twice in PZM 3 and cultured for 6 days in 4-well dishes in 1 mL of PZM 3 medium at 38.5 °C under a 5% CO_2_ atmosphere. After 40 h, the cleavage of embryos was assessed and after 144 h, the ability of embryos to reach the blastocyst stage was analyzed.

### 4.5. Immunoblotting

At the selected culture time, groups of 25 COCs were washed in PBS and solubilized in a Laemmli buffer containing 2% sodium dodecyl sulphate (SDS) and 5% 2-mercaptoethanol. Samples were boiled at 100 °C for 3 min and stored frozen at −20 °C. Subsequently, proteins were separated in 10% acrylamide/SDS gels and transferred to Immobilon-P membranes (Millipore, Bedford, MA, USA). Membranes were blocked in 5% low-fat dry milk in Tris-buffered saline (TBS) with 0.5% Tween 20 for 2 h at room temperature, and then incubated with a primary antibody diluted 1:1000 in 5% BSA in TBS-Tween, at 4°C overnight. The primary antibodies were p-ERK and ERK1 (detecting MAPK3/1), both from Santa Cruz Biotechnology (Santa Cruz, CA, USA) and pAKT and AKT, both from Cell Signaling Technology (Danvers, MA, USA). The secondary antibodies (Amersham ECL anti-mouse or anti-rabbit IgG, GE Healthcare, Little Chalfont, UK) were diluted 1:5000 in 2% BSA in TBS-Tween. The membranes were incubated with the secondary antibody for 1 h at room temperature, and then washed intensively in TBS-Tween. The immune reaction was detected by enhanced chemiluminescence (Pierce, Rockford, IL, USA) according to the manufacturer’s instructions. The intensity of the specific bands on the blots was analyzed by scanning densitometry using the free software Image J Version 1.29 (National Institute of Mental Health, Bethesda MD, USA).

### 4.6. Expression Analysis of Predicted Target and Quality-Related Genes Using RT-qPCR

A group of genes related to MAPK3/1 and AKT signaling was analyzed in CCs cultured in control and FLI medium for 4 or 8 h. The cumulus expansion-related genes were analyzed, based on our previous experience [13,31,40] at 4 h of culture and the signaling-, apoptosis, proliferation and cell communication, translation and mitochondria activity-related genes, based on our preliminary experiments, at 8 h of culture.

CCs were separated from oocytes by gentle pipetting and total RNA was extracted using an RNeasy Micro Kit (Qiagen, Hilden, Germany) with DNase-digestion treatment in the column. RNA was eluted in 25 uL of RNase-free water and its concentration and quality were assessed in a Nanodrop 1000. Template RNA in all samples was diluted to 10 ng/uL with RNase-free water, aliquoted and stored at −80 °C until analysis.

Primers for the selected genes were designed using the software Beacon Designer v. 8.21 and listed in Table 6. A one-step RT-qPCR was performed in a RotorGene 3000 cycler (Corbett Research, Mortlake, Austria) using the QIAGEN OneStep RT-PCR Kit (Qiagen) in a 20 µL reaction mixture containing 4 µL 5 X reaction buffer, 0.8 µL dNTP mix (10 nM stock), 0.4 µL forward and reverse primers (20 nM stock), 0.125 µL RNasine (20 IU/mL stock, Promega, Madison, WI, USA), 0.8 µL enzyme mix, 0.8 µL EvaGreen (Biotium, San Francisco, CA, USA), 3 µL RNA, and nuclease-free water. The reaction conditions were as follows: reverse transcription at 50 °C for 30 min, initial denaturation at 95 °C for 15 min, followed by PCR cycles consisting of denaturation at 95 °C for 15 s, annealing at a temperature specific for each set of primers (Table 6) for 15 s and extension at 72 °C for 20 s; and a final extension at 72 °C for 5 min. Fluorescence data were acquired at the end of each extension step. Products were verified by melting analysis and gel electrophoresis on 1.5% agarose gel with MidoriGreen Direct (Nippon Genetics, Dueren, Germany). Comparative analysis software (Corbett Research) was used for gene expression analyses after normalization to the geometric mean of *TBP* and *TUBA*1*B* mRNA abundance as internal control genes.

### 4.7. Statistical Analysis

The statistical analyses were performed with the software GraphPad Prism 5.0 (La Jolla, CA, USA). Each experiment was performed in at least 3 replicates. The densitometrical quantifications of MAPK3/1 and AKT were compared by analysis of variance (ANOVA) followed by Tukey’s multiple comparison post-test. The differences between kinase activities in control and FLI medium were analyzed by a *t*-test. Oocyte maturation and the development of embryos were analyzed by one-way ANOVA or a *t*-test, depending on the number of groups. Transcript levels were compared using *t*-test. Error bars indicate the standard error of the mean (SEM). Probability values <0.05 were considered to be statistically significant.

## 5. Conclusions

This study confirmed the enhanced meiotic and developmental competence of porcine oocytes cultured in a medium supplemented with FGF2, LIF and IGF1 (FLI medium), when compared with a control medium supplemented with gonadotropins and EGF. In the COCs cultured in FLI medium, the assessment of maturation kinetics revealed an accelerated oocyte maturation beyond 16 h of culture, robust activation of MAPK3/1 in CCs during the first hour of culture, and, for the first time, a higher activity of AKT by 16 h of culture followed by rapid deregulation by the end of the culture period. The expression of genes controlled by both kinases were also modulated in the FLI medium, including the genes that are considered to be markers of oocyte competence. The presented data indicate the involvement of both MAPK3/1 and AKT in mechanisms promoting the meiotic and developmental competence of porcine oocytes cultured in FLI medium. These data also demonstrate that the prompt activation of MAPK3/1 and high AKT activity during the first half of in vitro culture are molecular markers associated with the high quality of the produced pig oocytes.

## Figures and Tables

**Table 1:** Effect of gonadotropins, EGF and FLI cytokines on maturation of porcine oocytes in vitro.

Type of	No. of Oocytes	% of Oocytes in		
Medium	Examined	GV	GVBD	MII
M199+BSA	154	63.65 ± 5.75 ^A^	9.18 ± 0.94 ^AB^	26.95 ± 5.79 ^A^
+PMSG+hCG	150	63.58 ± 6.93 ^A^	10.38 ± 3.55 ^AB^	26.08 ± 5.03 ^A^
+PMSG+hCG+EGF (Control)	146	13.63 ± 6.45 ^B^	16.53 ± 2.38 ^A^	69.90 ± 4.05 ^B^
FLI	163	1.20 ± 0.69 ^B^	1.3 ± 0.76 ^B^	97.5 ± 0.14 ^C^

Data are expressed in percentages ± SEM. Values with different superscripts or no common letter in the superscript within a column are significantly different. GV: germinal vesicle; GVBD: germinal vesicle breakdown; MII: metaphase II.

**Table 2:** Effect of FGF2, LIF and IGF1 on maturation of porcine oocytes in vitro.

Type of	No. of Oocytes	% of Oocytes in		
Medium	Examined	GV	GVBD	MII
Control	91	22.11 ± 4.86 ^A^	12.41 ± 3.16	65.48 ± 3.12 ^A^
+FGF2	97	0 ± 0 ^B^	8.03 ± 2.93	91.97 ± 2.94 ^B^
+IGF1	97	4.80 ± 2.64 ^B^	4.13 ± 2.29	91.07 ± 4.47 ^B^
+LIF	92	0 ± 0 ^B^	4.67 ± 2.65	95.33 ± 2.45 ^B^
FLI	87	0 ± 0 ^B^	3.57 ± 0.50	96.43 ± 0.50 ^B^

Control: M199 supplemented with PMSG, hCG and EGF. FLI: Control medium supplemented with FGF2, LIF and IGF1. GV: germinal vesicle; GVBD: germinal vesicle breakdown; MII: metaphase II. Data are expressed in percentages ± SEM. Values with different superscripts within the column are significantly different (*P* < 0.01 for GV values; *P* < 0.001 for MII values).

**Table 3:** Effect of FLI medium on the course of maturation of porcine oocytes in vitro.

Type of	Time of	No. of Oocytes	% of Oocytes in		
Medium	Culture (h)	Examined	GV	GVBD	MII
Control	16	86	86.63 ± 5.95 ^A^	13.38 ± 5.95 ^A^	0 ± 0 ^A^
FLI	16	81	81.48 ± 6.25 ^A^	18.53 ± 6.25 ^A^	0 ± 0 ^A^
Control	24	97	71.93 ± 6.76 ^A^	22.40 ± 4.44 ^A^	5.68 ± 2.44 ^A^
FLI	24	99	30.83 ± 6.10 ^B^	56.03 ± 2.53 ^B^	13.15 ± 5.74 ^A^
Control	44	89	13.40 ± 6.61 ^A^	18.55 ± 7.45 ^A^	68.05 ± 5.72 ^A^
FLI	44	92	0 ± 0 ^A^	4.63 ± 2.67 ^A^	95.38 ± 2.67 ^B^

Data are expressed in percentages ± SEM. Values with different superscripts within the same interval of culture are significantly different (*P* < 0.05 for GV and MII values; P < 0.001 for GVBD value). GV: germinal vesicle; GVBD: germinal vesicle breakdown; MII: metaphase II.

**Table 4:** Development of porcine oocytes cultured in control and FLI medium after parthenogenetic activation.

Type of Medium	No. of Activated Oocytes	Cleavage Rate % ± SEM	Blastocyst Rate % ± SEM
Control	95	70.51 ± 1.39 ^A^	20.69 ± 3.45 ^A^
FLI	100	87.88 ± 1.99 ^B^	34.07 ± 0.38 ^B^

Data are expressed in percentages ± SEM. Values with different superscripts within the column are significantly different (*P* < 0.01 for cleavage rate; *P* < 0.05 for blastocyst rate).

**Table 5:** The composition of the control and FLI medium.

Component	Supplier	Control Medium	FLI Medium
TCM199	Sigma, M7528	TCM199	TCM199
Sodium pyruvate	Sigma, P4562	0.2 mM	0.2 mM
L-glutamin	Sigma, G8540	6.85 mM	6.85 mM
Cysteine	Sigma, C7352	0.57 mM	0.57 mM
Gentamycin	Roth, 0233	50 µg/mL	50 µg/mL
BSA	Sigma, A7030	1 mg/mL	1 mg/mL
PMSG	Prospec ^1^, HOR-272	10 IU/mL	10 IU/mL
hCG	Prospec ^1^, HOR-250	10 IU/mL	10 IU/mL
EGF	PeproTech ^2^, AF-100-15	10 ng/mL	10 IU/mL
human LIF	Merck ^3^, LIF1005	−	2 µL/mlL
human IGF1	PeproTech ^2^, AF-100-11	−	20 ng/mL
human FGF2	Sigma, F0291	−	40 ng/mL

^1^ Prospec, Rehovot, Israel; ^2^ PeproTech, London, England; ^3^ Merck Life Science, Prague, Czech Republic.

**Table 6:** List of primers used for RT-qPCR.

Gene	Amplicon Length (bp)	Sequence 5´–3´	Gene Accession No.	T_an_
*AKT1*	157	TAC TCC TTC CAG ACC CAC GA CGG AGT GCA GGT AGT CCA AG	NM_001159776.1	53
*ATP6*	141	AAT TCC TAT GCT CGT AAT ATG TTG AGT AGT GCT AAT	NC_000845	57
*BAX*	251	AAG CGC ATT GGA GAT GAA CT CGA TCT CGA AGG AAG TCC AG	XM_003127290.5	55
*BCL2L1*	196	GAA ACC CCT AGT GCC ATC AA GGG ACG TCA GGT CAC TGA AT	XM_021077292.1	55
*CAT*	128	CAA GAT TCT CCT GTG CTA CCC TAA CCT TCA CTT ACC	NM_214301.2	56
*CCND2*	116	CAG TGC TCC TAC TTC AAG ACC TCT TCT TCA CAC TTC	NM_214088.1	58
*CD44*	218	GAG GCG GCC CTG AAC ATA AAG GTA TTA GGC AGG TCT GTG AC	XM_013994425.2	58
*CDKN1B*	140	AAG ACT GAT GCA CCG GAC AG TTC GGG GAA CCG TCT GAA AC	NM_214316.1	53
*EIF4E*	148	GAATCTAATCAGGAGGTT AGTCTTCAACAGTATCAA	XM_003129314.3	53
*ERBB1*	241	CCC TCA AGG AGA TCA GCG AC CGC GGC TAA AGT TTC GAC AG	NM_214007	53
*EREG*	129	ATG GCT ACT GTT TGC ACG GA TGC TCA GAG GTT GTT GGA CG	XM_013978775.2	58
*FOXO1*	149	ATG GAG ACA CTT TGG ATT TAC TTC AAA TTA TCT GAC AG	NM_214014.3	53
*FOXO3*	113	ATT ATC CGT AGC GAA CTC AT TGC TTA GCA CCA GTG AAG	NM_001135959.1	57
*GJA1*	227	AGT GAT CCT TAC CAC GCC AC CGA TTC TGC TCG GCA CTG TA	NM_001244212.1	57
*GPX1*	127	GGT CTC CAG TGT GTC GCA AT GCT TCG ATG TCA GGC TCG AT	NM_214201.1	56
*HAS2*	407	GAA GTC ATG GGC AGG GAC AAT TC TGG CAG GCC CTT TCT ATG TTA	NM_214053.1	54
*JAK1*	195	GAC CGT CAC CTG CTT TGA GA ACG AAG CTG ATG TTG TCC GT	NM_214114.1	54
*MAPK1*	157	TAA GGT GCC ATG GAA CAG GC GGG CTC ATC ACT TGG GTC AT	NM_001198922.1	55
*MTOR*	151	CGA TGG CCA GGG ATC TCT TC TCG GCC AAG TTT AAG AGC GT	XM_003127584.6	53
*PANX1*	159	CGC GCA GGA AAT CTC AAT CG TTA TGC AGG CAC AGT GGG AG	XM_003482597.4	57
*PCNA*	153	TAA TGC AGA CAC CTT GGC ACT GCA AAT TCA CCA GAA GGC ATC	NM_001291925.1	55
*POLG*	152	ACT GGC TGG ACA TCA GCA GT ACA GTA CCG CAT CAG GTC C	XM_001927064.5	55
*PTX3*	208	CGC CAA TAC TGT GAT TTC C TAT TTC ATC AAA GCC ACC AC	NM_001244783.1	54
*SOD1*	139	AAC ATG GTG GGC CAA AGG AT GTG CGG CCA ATG ATG GAA TG	NM_001190422.1	55
*SOD2*	220	GGT GGA GGC CAC ATC AAT CA AAC AAG CGG CAA TCT GCA AG	NM_214127.2	55
*STAT3*	139	CTT GCC AGT CGT GGT CAT CT CAC TTG ATC CCA CGT TCC GA	NM_001044580.1	57
*TNFAIP6*	119	TAT ACG ACA GTT ACG ACG AC GGA AGC ATC ACT TAG GAA T	NM_001159607.1	54
*TBP*	115	ATA GCC TTC CAC CTT ACG CTC ATA GGC TGT GGA GTC AGT CCT	XM_021085483.1	58
*TUBA1B*	130	AGT TTT CTG AGG CCC GTG AG TGC AGG GCT TAA AGG AAT GGT	NM_001044544.1	58

T_an_: annealing temperature (°C).

## Data Availability

The data underlying this article are presented in the article.
